# Correction: Divalent cation and chloride ion sites of chicken acid sensing ion channel 1a elucidated by x-ray crystallography

**DOI:** 10.1371/journal.pone.0209147

**Published:** 2018-12-10

**Authors:** Nate Yoder, Eric Gouaux

The following information is missing from the Funding section: The NE-CAT beamlines are funded by the NIH (NIGMS grant P41 GM103403 and NIH-ORIP HEI grant S10 RR029205), and used resources of the Advanced Photon Source at Argonne National Laboratory under DOE Contract No. DE-AC02-06CH11357.
