# Supplementary material for: Divalent cation and chloride ion sites of chicken acid sensing ion channel 1a elucidated by x-ray crystallography
Source: PLoS One. 2018 Aug 29;13(8):e0202134. doi: 10.1371/journal.pone.0202134 (PMC6114778; doi:10.1371/journal.pone.0202134)
Supplement: S1 Fig — (PDF) [file pone.0202134.s001.pdf]

Full wwPDB X-ray Structure Validation Report (i)

Aug 23, 2017 – 11:25 AM EDT

PDB ID	:	5WKX
Title	:	Barium sites in the structure of a resting acid sensing ion channel
Deposited on	:	2017-07-25
Resolution	:	4.03 Å(reported)

This is a Full wwPDB X-ray Structure Validation Report.

This report is produced by the wwPDB biocuration pipeline after annotation of the structure.

We welcome your comments at *validation@mail.wwpdb.org*
A user guide is available at
http://wwpdb.org/validation/2016/XrayValidationReportHelp
with specific help available everywhere you see the (i) symbol.

The following versions of software and data (see references (i)) were used in the production of this report:

MolProbity	:	4.02b-467
Mogul	:	1.7.2 (RC1), CSD as538be (2017)
Xtriage (Phenix)	:	1.9-1692
EDS	:	rb-20029824
Percentile statistics	:	20161228.v01 (using entries in the PDB archive December 28th 2016)
Refmac	:	5.8.0135
CCP4	:	6.5.0
Ideal geometry (proteins)	:	Engh & Huber (2001)
Ideal geometry (DNA, RNA)	:	Parkinson et al. (1996)
Validation Pipeline (wwPDB-VP)	:	rb-20029824

CONFIDENTIAL VALIDATION REPORT

1 Overall quality at a glance

The following experimental techniques were used to determine the structure:
X-RAY DIFFRACTION

The reported resolution of this entry is 4.03 Å.

Percentile scores (ranging between 0-100) for global validation metrics of the entry are shown in the following graphic. The table shows the number of entries on which the scores are based.

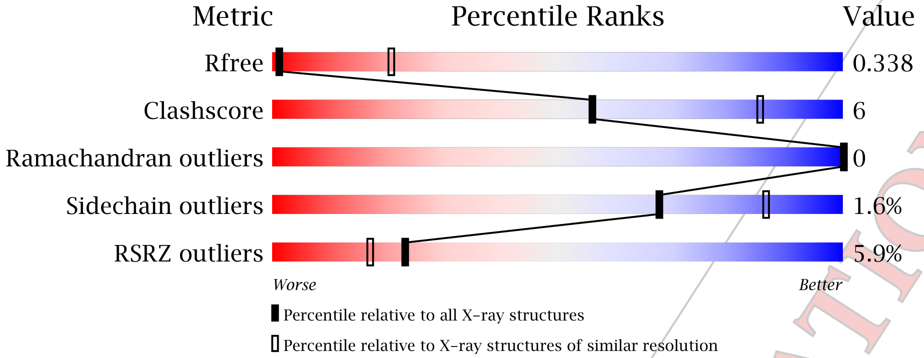

Metric	Whole archive (#Entries)	Similar resolution (#Entries, resolution range(Å))
R_{free}	100719	1094 (4.48-3.60)
Clashscore	112137	1194 (4.48-3.60)
Ramachandran outliers	110173	1145 (4.48-3.60)
Sidechain outliers	110143	1132 (4.48-3.60)
RSRZ outliers	101464	1105 (4.48-3.60)

The table below summarises the geometric issues observed across the polymeric chains and their fit to the electron density. The red, orange, yellow and green segments on the lower bar indicate the fraction of residues that contain outliers for >=3, 2, 1 and 0 types of geometric quality criteria. A grey segment represents the fraction of residues that are not modelled. The numeric value for each fraction is indicated below the corresponding segment, with a dot representing fractions <=5% The upper red bar (where present) indicates the fraction of residues that have poor fit to the electron density. The numeric value is given above the bar.

Mol	Chain	Length	Quality of chain
1	A	439	3%<br>78% 16% • 5%
1	B	439	3%<br>83% 12% 5%
1	C	439	10%<br>82% 12% 6%

CONFIDENTIAL VALIDATION REPORT

2 Entry composition (i)

There are 4 unique types of molecules in this entry. The entry contains 9355 atoms, of which 0 are hydrogens and 0 are deuteriums.

In the tables below, the ZeroOcc column contains the number of atoms modelled with zero occupancy, the AltConf column contains the number of residues with at least one atom in alternate conformation and the Trace column contains the number of residues modelled with at most 2 atoms.

- Molecule 1 is a protein called Acid-sensing ion channel 1.

Mol	Chain	Residues	Atoms					ZeroOcc	AltConf	Trace
1	A	417	Total 3140	C 2019	N 515	O 579	S 27	0	0	0
1	C	414	Total 3012	C 1933	N 494	O 559	S 26	0	0	0
1	B	418	Total 3115	C 2000	N 508	O 580	S 27	0	0	0

- Molecule 2 is N-ACETYL-D-GLUCOSAMINE (three-letter code: NAG) (formula: $C_8H_{15}NO_6$).

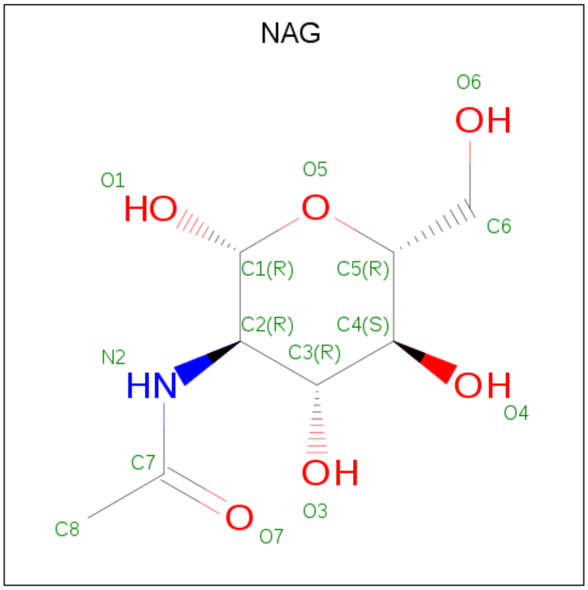

Mol	Chain	Residues	Atoms				ZeroOcc	AltConf
2	A	1	Total 14	C 8	N 1	O 5	0	0
2	C	1	Total 14	C 8	N 1	O 5	0	0
2	C	1	Total 14	C 8	N 1	O 5	0	0

Continued on next page...

CONFIDENTIAL VALIDATION REPORT

Continued from previous page...

Mol	Chain	Residues	Atoms	ZeroOcc	AltConf
2	B	1	Total C N O<br>14 8 1 5	0	0
2	B	1	Total C N O<br>14 8 1 5	0	0
2	B	1	Total C N O<br>14 8 1 5	0	0

- Molecule 3 is CHLORIDE ION (three-letter code: CL) (formula: Cl).

Mol	Chain	Residues	Atoms	ZeroOcc	AltConf
3	B	1	Total Cl<br>1 1	0	0
3	A	1	Total Cl<br>1 1	0	0
3	C	1	Total Cl<br>1 1	0	0

- Molecule 4 is BARIUM ION (three-letter code: BA) (formula: Ba).

Mol	Chain	Residues	Atoms	ZeroOcc	AltConf
4	A	1	Total Ba<br>1 1	0	0

CONFIDENTIAL VALIDATION REPORT

3 Residue-property plots (i)

These plots are drawn for all protein, RNA and DNA chains in the entry. The first graphic for a chain summarises the proportions of the various outlier classes displayed in the second graphic. The second graphic shows the sequence view annotated by issues in geometry and electron density. Residues are color-coded according to the number of geometric quality criteria for which they contain at least one outlier: green = 0, yellow = 1, orange = 2 and red = 3 or more. A red dot above a residue indicates a poor fit to the electron density (RSRZ > 2). Stretches of 2 or more consecutive residues without any outlier are shown as a green connector. Residues present in the sample, but not in the model, are shown in grey.

- Molecule 1: Acid-sensing ion channel 1

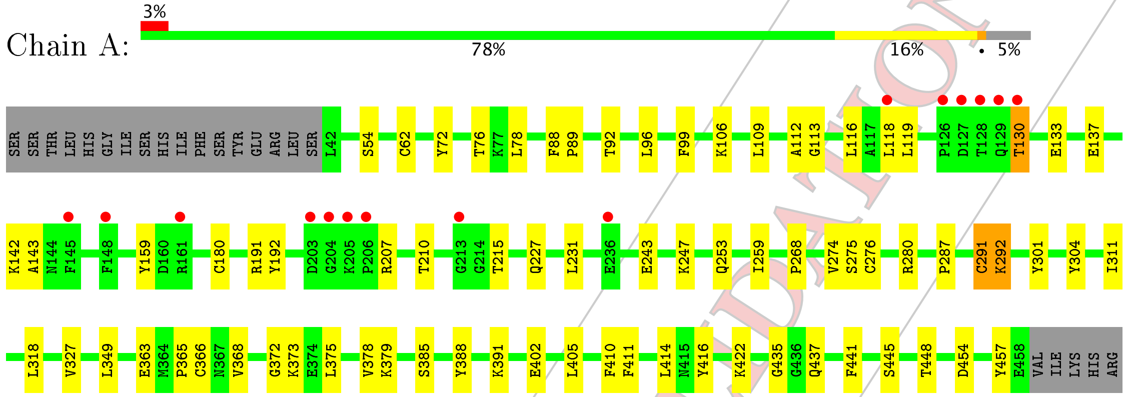

- Molecule 1: Acid-sensing ion channel 1

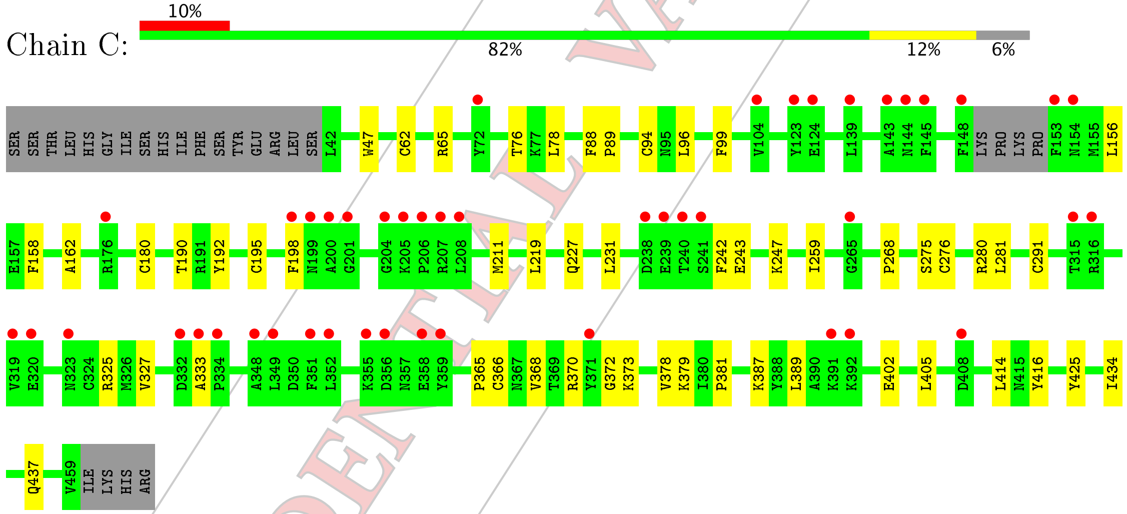

- Molecule 1: Acid-sensing ion channel 1

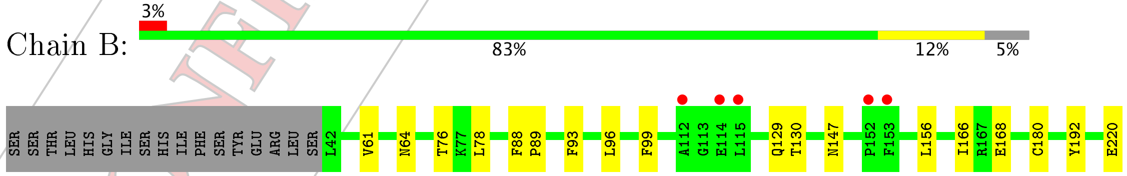

CONFIDENTIAL VALIDATION REPORT

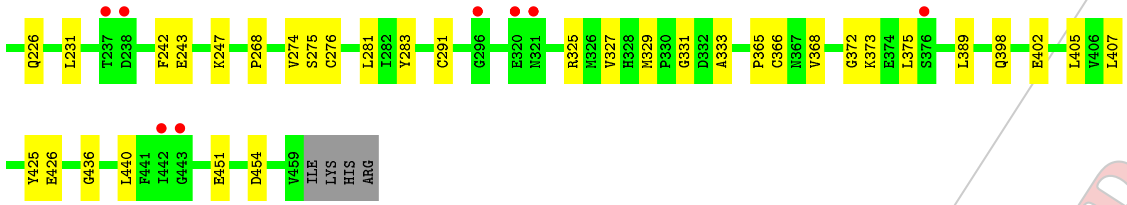

CONFIDENTIAL VALIDATION REPORT

4 Data and refinement statistics (i)

Property	Value	Source
Space group	P 21 21 21	Depositor
Cell constants<br>a, b, c, α, β, γ	108.01Å 126.30Å 156.32Å<br>90.00° 90.00° 90.00°	Depositor
Resolution (Å)	49.66 – 4.03<br>49.66 – 4.03	Depositor<br>EDS
% Data completeness<br>(in resolution range)	99.4 (49.66-4.03)<br>99.5 (49.66-4.03)	Depositor<br>EDS
R_{merge}	(Not available)	Depositor
R_{sym}	(Not available)	Depositor
$< I/\sigma(I) >$ [1]	1.70 (at 4.00Å)	Xtriage
Refinement program	PHENIX (dev_2597: ???)	Depositor
R, R_{free}	0.302 , 0.313<br>0.323 , 0.338	Depositor<br>DCC
R_{free} test set	905 reflections (5.26%)	DCC
Wilson B-factor (Å^2)	193.1	Xtriage
Anisotropy	0.490	Xtriage
Bulk solvent k_{sol}(e/Å^3), B_{sol}(Å^2)	0.25 , 66.6	EDS
L-test for twinning[2]	$< \|L\| >$ = 0.39, $< L^2 >$ = 0.22	Xtriage
Estimated twinning fraction	No twinning to report.	Xtriage
F_o,F_c correlation	0.80	EDS
Total number of atoms	9355	wwPDB-VP
Average B, all atoms (Å^2)	141.0	wwPDB-VP

Xtriage's analysis on translational NCS is as follows: *The largest off-origin peak in the Patterson function is 3.16% of the height of the origin peak. No significant pseudotranslation is detected.*

[1] Intensities estimated from amplitudes.

[2] Theoretical values of $< |L| >$, $< L^2 >$ for acentric reflections are 0.5, 0.333 respectively for untwinned datasets, and 0.375, 0.2 for perfectly twinned datasets.

CONFIDENTIAL VALIDATION REPORT

5 Model quality (i)

5.1 Standard geometry (i)

Bond lengths and bond angles in the following residue types are not validated in this section: CL, BA, NAG

The Z score for a bond length (or angle) is the number of standard deviations the observed value is removed from the expected value. A bond length (or angle) with $|Z| > 5$ is considered an outlier worth inspection. RMSZ is the root-mean-square of all Z scores of the bond lengths (or angles).

Mol	Chain	Bond lengths		Bond angles	
		RMSZ	#$|Z|$ >5	RMSZ	#$|Z|$ >5
1	A	0.31	0/3215	0.50	0/4376
1	B	0.31	0/3189	0.50	0/4349
1	C	0.28	0/3081	0.48	0/4206
All	All	0.30	0/9485	0.49	0/12931

There are no bond length outliers.

There are no bond angle outliers.

There are no chirality outliers.

There are no planarity outliers.

5.2 Too-close contacts (i)

In the following table, the Non-H and H(model) columns list the number of non-hydrogen atoms and hydrogen atoms in the chain respectively. The H(added) column lists the number of hydrogen atoms added and optimized by MolProbity. The Clashes column lists the number of clashes within the asymmetric unit, whereas Symm-Clashes lists symmetry related clashes.

Mol	Chain	Non-H	H(model)	H(added)	Clashes	Symm-Clashes
1	A	3140	0	2895	51	6
1	B	3115	0	2836	34	6
1	C	3012	0	2680	34	0
2	A	14	0	13	0	0
2	B	42	0	38	0	0
2	C	28	0	26	0	0
3	A	1	0	0	1	0
3	B	1	0	0	0	0
3	C	1	0	0	0	0
4	A	1	0	0	0	0
All	All	9355	0	8488	104	6

CONFIDENTIAL VALIDATION REPORT

The all-atom clashscore is defined as the number of clashes found per 1000 atoms (including hydrogen atoms). The all-atom clashscore for this structure is 6.

All (104) close contacts within the same asymmetric unit are listed below, sorted by their clash magnitude.

Atom-1	Atom-2	Interatomic distance (Å)	Clash overlap (Å)
1:A:191:ARG:HH22	1:A:349:LEU:HD13	1.45	0.81
1:A:192:TYR:HE2	1:A:247:LYS:HD3	1.53	0.73
1:C:96:LEU:HD13	1:C:243:GLU:HG3	1.72	0.71
1:A:291:CYS:SG	1:A:292:LYS:N	2.64	0.71
1:A:133:GLU:O	1:A:137:GLU:HG2	1.95	0.66
1:B:192:TYR:HE2	1:B:247:LYS:HD3	1.63	0.64
1:C:192:TYR:HE2	1:C:247:LYS:HD3	1.61	0.63
1:A:365:PRO:HG2	1:A:368:VAL:HG22	1.81	0.62
1:B:276:CYS:HA	1:B:372:GLY:O	1.99	0.62
1:C:275:SER:O	1:C:373:LYS:HA	1.99	0.62
1:A:96:LEU:HD13	1:A:243:GLU:HG3	1.80	0.62
1:C:325:ARG:HD3	1:C:333:ALA:HB3	1.83	0.61
1:B:192:TYR:CE2	1:B:247:LYS:HD3	2.34	0.61
1:A:99:PHE:CE2	1:A:231:LEU:HD21	2.36	0.60
1:B:130:THR:HG22	1:B:130:THR:O	2.00	0.59
1:A:76:THR:HG21	1:C:78:LEU:HD12	1.84	0.59
1:A:78:LEU:HD12	1:B:76:THR:HG21	1.85	0.58
1:A:276:CYS:HA	1:A:372:GLY:O	2.03	0.58
1:C:276:CYS:HA	1:C:372:GLY:O	2.04	0.57
1:B:275:SER:O	1:B:373:LYS:HA	2.06	0.56
1:B:325:ARG:HH22	1:B:331:GLY:C	2.09	0.55
1:A:378:VAL:HG11	1:B:96:LEU:HD11	1.87	0.55
1:C:76:THR:HG21	1:B:78:LEU:HD12	1.88	0.55
1:A:275:SER:O	1:A:373:LYS:HA	2.07	0.55
1:A:422:LYS:NZ	3:A:502:CL:CL	2.77	0.55
1:C:192:TYR:CE2	1:C:247:LYS:HD3	2.43	0.54
1:C:156:LEU:HD11	1:C:327:VAL:HA	1.90	0.53
1:C:365:PRO:HG2	1:C:368:VAL:HG22	1.90	0.53
1:A:96:LEU:HD11	1:C:378:VAL:HG11	1.91	0.53
1:A:99:PHE:CE1	1:A:116:LEU:HD22	2.44	0.53
1:C:99:PHE:CZ	1:C:231:LEU:HD21	2.45	0.52
1:A:304:TYR:OH	1:A:363:GLU:O	2.17	0.51
1:A:113:GLY:HA3	1:A:119:LEU:HD12	1.93	0.51
1:A:227:GLN:NE2	1:A:402:GLU:O	2.43	0.51
1:B:451:GLU:O	1:B:454:ASP:HB3	2.11	0.51
1:C:158:PHE:O	1:C:162:ALA:HB3	2.11	0.50
1:A:268:PRO:HA	1:A:405:LEU:HB3	1.93	0.50

Continued on next page...

CONFIDENTIAL VALIDATION REPORT

Continued from previous page...

Atom-1	Atom-2	Interatomic distance (Å)	Clash overlap (Å)
1:B:156:LEU:HD12	1:B:327:VAL:HG12	1.93	0.50
1:C:190:THR:OG1	1:C:195:CYS:SG	2.70	0.50
1:B:99:PHE:CZ	1:B:231:LEU:HD21	2.47	0.50
1:C:227:GLN:NE2	1:C:402:GLU:O	2.45	0.50
1:A:88:PHE:CG	1:A:89:PRO:HD2	2.48	0.49
1:B:281:LEU:HD23	1:B:283:TYR:OH	2.13	0.49
1:A:385:SER:HB2	1:B:242:PHE:HD1	1.77	0.49
1:A:268:PRO:HD2	1:C:379:LYS:HB2	1.95	0.49
1:A:118:LEU:HD21	1:A:231:LEU:HD22	1.95	0.49
1:A:210:THR:HG21	1:A:411:PHE:HD2	1.77	0.48
1:A:192:TYR:CE2	1:A:247:LYS:HD3	2.40	0.48
1:A:379:LYS:HB2	1:B:268:PRO:HD2	1.94	0.48
1:B:88:PHE:CG	1:B:89:PRO:HD2	2.48	0.48
1:A:109:LEU:HD23	1:A:143:ALA:HB2	1.96	0.47
1:A:130:THR:HG22	1:C:387:LYS:HB3	1.97	0.47
1:C:414:LEU:HD23	1:C:414:LEU:HA	1.74	0.47
1:C:88:PHE:CG	1:C:89:PRO:HD2	2.50	0.47
1:B:220:GLU:HA	1:B:407:LEU:O	2.15	0.46
1:C:198:PHE:CD2	1:C:219:LEU:HD22	2.51	0.46
1:A:365:PRO:HG2	1:A:368:VAL:CG2	2.45	0.45
1:A:445:SER:H	1:A:448:THR:HB	1.81	0.45
1:A:435:GLY:HA3	1:B:436:GLY:O	2.16	0.45
1:A:388:TYR:N	1:B:130:THR:HG21	2.31	0.45
1:C:268:PRO:HA	1:C:405:LEU:HB3	1.99	0.45
1:B:268:PRO:HA	1:B:405:LEU:HB3	1.98	0.44
1:C:280:ARG:HD2	1:C:416:TYR:CD2	2.52	0.44
1:A:112:ALA:O	1:A:116:LEU:HG	2.17	0.44
1:C:62:CYS:HA	1:C:437:GLN:NE2	2.32	0.44
1:B:398:GLN:O	1:B:402:GLU:HG2	2.17	0.44
1:B:61:VAL:O	1:B:64:ASN:HB3	2.17	0.44
1:B:93:PHE:HZ	1:B:166:ILE:HD13	1.82	0.44
1:B:325:ARG:NH2	1:B:333:ALA:H	2.15	0.44
1:A:280:ARG:HD2	1:A:416:TYR:CD1	2.53	0.44
1:A:72:TYR:CD1	1:A:287:PRO:HG2	2.53	0.43
1:B:96:LEU:HD13	1:B:243:GLU:HG3	2.00	0.43
1:C:94:CYS:SG	1:C:259:ILE:HG21	2.57	0.43
1:B:168:GLU:HB3	1:B:226:GLN:NE2	2.34	0.43
1:B:365:PRO:HG2	1:B:368:VAL:HG22	1.99	0.43
1:C:94:CYS:SG	1:C:259:ILE:HD13	2.59	0.43
1:B:325:ARG:CG	1:B:329:MET:HB2	2.48	0.43
1:A:62:CYS:HA	1:A:437:GLN:NE2	2.35	0.42

Continued on next page...

CONFIDENTIAL VALIDATION REPORT

Continued from previous page...

Atom-1	Atom-2	Interatomic distance (Å)	Clash overlap (Å)
1:A:227:GLN:HE21	1:A:227:GLN:HB2	1.62	0.42
1:A:54:SER:OG	1:A:441:PHE:O	2.34	0.42
1:B:274:VAL:HG22	1:B:375:LEU:CD2	2.49	0.42
1:C:281:LEU:HD21	1:C:370:ARG:HH21	1.85	0.42
1:C:381:PRO:HB3	1:C:389:LEU:HD12	2.00	0.42
1:C:434:ILE:HA	1:C:437:GLN:NE2	2.34	0.42
1:C:211:MET:C	1:C:414:LEU:HD11	2.41	0.42
1:A:99:PHE:CZ	1:A:231:LEU:HD11	2.55	0.41
1:C:280:ARG:HD2	1:C:416:TYR:CE2	2.55	0.41
1:A:92:THR:CG2	1:A:259:ILE:HD11	2.50	0.41
1:A:215:THR:HA	1:A:410:PHE:CD1	2.56	0.41
1:C:242:PHE:CE2	1:B:389:LEU:HD21	2.56	0.41
1:A:301:TYR:CE2	1:A:311:ILE:HD11	2.56	0.41
1:A:318:LEU:HD23	1:A:318:LEU:HA	1.85	0.41
1:A:414:LEU:HD23	1:A:414:LEU:HA	1.91	0.41
1:C:227:GLN:HB2	1:C:227:GLN:HE21	1.70	0.40
1:A:274:VAL:HG22	1:A:375:LEU:CD2	2.51	0.40
1:A:435:GLY:HA3	1:B:440:LEU:HB2	2.02	0.40
1:A:391:LYS:HE2	1:B:129:GLN:HB3	2.03	0.40
1:C:65:ARG:NH1	1:B:426:GLU:OE1	2.53	0.40
1:A:454:ASP:O	1:A:457:TYR:HB3	2.21	0.40
1:B:129:GLN:HG3	1:B:129:GLN:H	1.71	0.40
1:A:159:TYR:HD2	1:A:327:VAL:HG11	1.86	0.40
1:A:106:LYS:HG3	1:A:142:LYS:O	2.21	0.40
1:A:207:ARG:CZ	1:A:253:GLN:HB2	2.51	0.40
1:C:247:LYS:O	1:C:259:ILE:HD11	2.22	0.40

All (6) symmetry-related close contacts are listed below. The label for Atom-2 includes the symmetry operator and encoded unit-cell translations to be applied.

Atom-1	Atom-2	Interatomic distance (Å)	Clash overlap (Å)
1:A:416:TYR:CE2	1:B:147:ASN:CB[4_555]	1.68	0.52
1:A:280:ARG:CZ	1:B:147:ASN:OD1[4_555]	1.81	0.39
1:A:416:TYR:CE2	1:B:147:ASN:CG[4_555]	1.92	0.28
1:A:416:TYR:OH	1:B:147:ASN:N[4_555]	2.16	0.04
1:A:280:ARG:NH2	1:B:147:ASN:OD1[4_555]	2.18	0.02
1:A:416:TYR:CD2	1:B:147:ASN:CG[4_555]	2.19	0.01

CONFIDENTIAL VALIDATION REPORT

5.3 Torsion angles (i)

5.3.1 Protein backbone (i)

In the following table, the Percentiles column shows the percent Ramachandran outliers of the chain as a percentile score with respect to all X-ray entries followed by that with respect to entries of similar resolution.

The Analysed column shows the number of residues for which the backbone conformation was analysed, and the total number of residues.

Mol	Chain	Analysed	Favoured	Allowed	Outliers	Percentiles	
1	A	415/439 (94%)	409 (99%)	6 (1%)	0	100	100
1	B	416/439 (95%)	408 (98%)	8 (2%)	0	100	100
1	C	410/439 (93%)	403 (98%)	7 (2%)	0	100	100
All	All	1241/1317 (94%)	1220 (98%)	21 (2%)	0	100	100

There are no Ramachandran outliers to report.

5.3.2 Protein sidechains (i)

In the following table, the Percentiles column shows the percent sidechain outliers of the chain as a percentile score with respect to all X-ray entries followed by that with respect to entries of similar resolution.

The Analysed column shows the number of residues for which the sidechain conformation was analysed, and the total number of residues.

Mol	Chain	Analysed	Rotameric	Outliers	Percentiles	
1	A	308/384 (80%)	303 (98%)	5 (2%)	68	86
1	B	303/384 (79%)	299 (99%)	4 (1%)	73	88
1	C	278/384 (72%)	273 (98%)	5 (2%)	64	85
All	All	889/1152 (77%)	875 (98%)	14 (2%)	68	86

All (14) residues with a non-rotameric sidechain are listed below:

Mol	Chain	Res	Type
1	A	130	THR
1	A	180	CYS
1	A	291	CYS
1	A	292	LYS
1	A	366	CYS

Continued on next page...

CONFIDENTIAL VALIDATION REPORT

Continued from previous page...

Mol	Chain	Res	Type
1	C	47	TRP
1	C	180	CYS
1	C	291	CYS
1	C	366	CYS
1	C	425	TYR
1	B	180	CYS
1	B	291	CYS
1	B	366	CYS
1	B	425	TYR

Some sidechains can be flipped to improve hydrogen bonding and reduce clashes. There are no such sidechains identified.

5.3.3 RNA (i)

There are no RNA molecules in this entry.

5.4 Non-standard residues in protein, DNA, RNA chains (i)

There are no non-standard protein/DNA/RNA residues in this entry.

5.5 Carbohydrates (i)

There are no carbohydrates in this entry.

5.6 Ligand geometry (i)

Of 10 ligands modelled in this entry, 4 are monoatomic - leaving 6 for Mogul analysis.

In the following table, the Counts columns list the number of bonds (or angles) for which Mogul statistics could be retrieved, the number of bonds (or angles) that are observed in the model and the number of bonds (or angles) that are defined in the chemical component dictionary. The Link column lists molecule types, if any, to which the group is linked. The Z score for a bond length (or angle) is the number of standard deviations the observed value is removed from the expected value. A bond length (or angle) with $|Z| > 2$ is considered an outlier worth inspection. RMSZ is the root-mean-square of all Z scores of the bond lengths (or angles).

Mol	Type	Chain	Res	Link	Bond lengths			Bond angles		
					Counts	RMSZ	$\#\|Z\| > 2$	Counts	RMSZ	$\#\|Z\| > 2$
2	NAG	A	501	1	14,14,15	0.24	0	15,19,21	0.46	0
2	NAG	B	501	1,2	14,14,15	0.24	0	15,19,21	0.49	0

CONFIDENTIAL VALIDATION REPORT

Mol	Type	Chain	Res	Link	Bond lengths			Bond angles		
					Counts	RMSZ	$\#\|Z\|>2$	Counts	RMSZ	$\#\|Z\|>2$
2	NAG	B	502	2	14,14,15	0.25	0	15,19,21	0.51	0
2	NAG	B	503	1	14,14,15	0.31	0	15,19,21	0.51	0
2	NAG	C	501	1	14,14,15	0.21	0	15,19,21	0.61	0
2	NAG	C	502	1	14,14,15	0.29	0	15,19,21	0.51	0

In the following table, the Chirals column lists the number of chiral outliers, the number of chiral centers analysed, the number of these observed in the model and the number defined in the chemical component dictionary. Similar counts are reported in the Torsion and Rings columns. '-' means no outliers of that kind were identified.

Mol	Type	Chain	Res	Link	Chirals	Torsions	Rings
2	NAG	A	501	1	-	0/6/23/26	0/1/1/1
2	NAG	B	501	1,2	-	0/6/23/26	0/1/1/1
2	NAG	B	502	2	-	0/6/23/26	0/1/1/1
2	NAG	B	503	1	-	0/6/23/26	0/1/1/1
2	NAG	C	501	1	-	0/6/23/26	0/1/1/1
2	NAG	C	502	1	-	0/6/23/26	0/1/1/1

There are no bond length outliers.

There are no bond angle outliers.

There are no chirality outliers.

There are no torsion outliers.

There are no ring outliers.

No monomer is involved in short contacts.

5.7 Other polymers (i)

There are no such residues in this entry.

5.8 Polymer linkage issues (i)

There are no chain breaks in this entry.

CONFIDENTIAL VALIDATION REPORT

6 Fit of model and data (i)

6.1 Protein, DNA and RNA chains (i)

In the following table, the column labelled '#RSRZ> 2' contains the number (and percentage) of RSRZ outliers, followed by percent RSRZ outliers for the chain as percentile scores relative to all X-ray entries and entries of similar resolution. The OWAB column contains the minimum, median, 95th percentile and maximum values of the occupancy-weighted average B-factor per residue. The column labelled 'Q< 0.9' lists the number of (and percentage) of residues with an average occupancy less than 0.9.

Mol	Chain	Analysed	<RSRZ>	#RSRZ>2			OWAB(Å^2)	Q<0.9
1	A	417/439 (94%)	-0.01	15 (3%)	43	34	70, 125, 200, 237	0
1	B	418/439 (95%)	-0.01	13 (3%)	49	39	76, 133, 203, 246	0
1	C	414/439 (94%)	0.46	46 (11%)	6	6	87, 153, 248, 270	0
All	All	1249/1317 (94%)	0.15	74 (5%)	23	17	70, 136, 222, 270	0

All (74) RSRZ outliers are listed below:

Mol	Chain	Res	Type	RSRZ
1	C	332	ASP	8.7
1	C	323	ASN	7.3
1	A	127	ASP	7.3
1	C	334	PRO	7.0
1	C	333	ALA	6.9
1	C	315	THR	6.4
1	C	145	PHE	6.2
1	C	206	PRO	5.9
1	A	130	THR	5.5
1	C	319	VAL	5.3
1	C	199	ASN	5.1
1	A	128	THR	5.0
1	B	237	THR	4.8
1	B	238	ASP	4.5
1	A	129	GLN	4.5
1	B	320	GLU	4.5
1	C	207	ARG	4.2
1	A	206	PRO	4.1
1	B	152	PRO	4.1
1	C	352	LEU	3.9
1	B	153	PHE	3.8
1	A	148	PHE	3.7
1	A	236	GLU	3.5

Continued on next page...

CONFIDENTIAL VALIDATION REPORT

Continued from previous page...

Mol	Chain	Res	Type	RSRZ
1	C	320	GLU	3.5
1	C	198	PHE	3.4
1	C	153	PHE	3.4
1	C	316	ARG	3.4
1	B	114	GLU	3.4
1	C	123	TYR	3.3
1	C	348	ALA	3.3
1	C	239	GLU	3.3
1	C	349	LEU	3.3
1	B	115	LEU	3.2
1	A	126	PRO	3.2
1	C	238	ASP	3.1
1	C	241	SER	3.1
1	C	201	GLY	3.0
1	C	144	ASN	3.0
1	C	139	LEU	3.0
1	C	148	PHE	2.9
1	A	204	GLY	2.9
1	C	351	PHE	2.8
1	C	355	LYS	2.8
1	B	376	SER	2.8
1	C	200	ALA	2.8
1	C	143	ALA	2.8
1	C	408	ASP	2.7
1	B	442	ILE	2.7
1	C	371	TYR	2.6
1	B	443	GLY	2.6
1	A	205	LYS	2.6
1	C	391	LYS	2.6
1	C	204	GLY	2.5
1	C	240	THR	2.5
1	C	104	VAL	2.5
1	B	112	ALA	2.5
1	A	203	ASP	2.5
1	C	205	LYS	2.4
1	C	124	GLU	2.4
1	C	176	ARG	2.3
1	A	118	LEU	2.3
1	B	321	ASN	2.3
1	C	208	LEU	2.3
1	A	213	GLY	2.3
1	C	265	GLY	2.3

Continued on next page...

CONFIDENTIAL VALIDATION REPORT

Continued from previous page...

Mol	Chain	Res	Type	RSRZ
1	B	296	GLY	2.3
1	C	72	TYR	2.2
1	A	161	ARG	2.2
1	C	392	LYS	2.2
1	A	145	PHE	2.2
1	C	358	GLU	2.2
1	C	154	ASN	2.1
1	C	359	TYR	2.0
1	C	356	ASP	2.0

6.2 Non-standard residues in protein, DNA, RNA chains (i)

There are no non-standard protein/DNA/RNA residues in this entry.

6.3 Carbohydrates (i)

There are no carbohydrates in this entry.

6.4 Ligands (i)

In the following table, the Atoms column lists the number of modelled atoms in the group and the number defined in the chemical component dictionary. LLDF column lists the quality of electron density of the group with respect to its neighbouring residues in protein, DNA or RNA chains. The B-factors column lists the minimum, median, 95th percentile and maximum values of B factors of atoms in the group. The column labelled 'Q< 0.9' lists the number of atoms with occupancy less than 0.9.

Mol	Type	Chain	Res	Atoms	RSCC	RSR	LLDF	B-factors($Å^2$)	Q<0.9
3	CL	A	502	1/1	0.87	0.81	-	120,120,120,120	0
3	CL	B	504	1/1	0.61	0.81	-	134,134,134,134	0
2	NAG	B	501	14/15	0.85	0.16	-	140,169,210,225	0
2	NAG	C	501	14/15	0.83	0.43	-	161,185,200,202	0
2	NAG	B	502	14/15	0.87	0.27	-	160,198,228,229	0
2	NAG	C	502	14/15	0.80	0.92	-	186,208,227,228	0
2	NAG	A	501	14/15	0.75	0.63	-	163,187,200,204	0
3	CL	C	503	1/1	0.88	0.25	-	165,165,165,165	0
2	NAG	B	503	14/15	0.88	0.41	-	159,190,201,216	0
4	BA	A	503	1/1	0.19	0.22	-	263,263,263,263	0

CONFIDENTIAL VALIDATION REPORT

6.5 Other polymers (i)

There are no such residues in this entry.

CONFIDENTIAL VALIDATION REPORT
